# Reduced-dose direct oral anticoagulants for the secondary prevention of venous thromboembolism: finding the sweet spot

**DOI:** 10.1016/j.rpth.2025.103170

**Published:** 2025-09-03

**Authors:** Benjamin Crichi, Maxime Delrue, Corinne Frere

**Affiliations:** 1 Department of Internal Medicine, Saint-Louis Hospital, Assistance Publique-Hôpitaux de Paris, Paris, France; 2 Laboratory of Hematology, Lariboisière Hospital, Assistance Publique-Hôpitaux de Paris, Université Paris Cité, INSERM UMR-S-1144, Paris, France; 3 Department of Vascular Medicine, Hôpitaux Paris Saint-Joseph et Marie-Lannelongue, Paris, France; 4 Department of Hematology, Pitié-Salpêtrière Hospital, Assistance Publique-Hôpitaux de Paris, Sorbonne Université, INSERM UMRS-1166, Institut hospitalo-Universitaire ICAN, Paris, France

Venous thromboembolism (VTE), including deep vein thrombosis and pulmonary embolism, is a common condition that requires anticoagulation for at least 3 to 6 months to prevent local thrombosis extension and/or recurrence [[1], [2], [3]]. After 3 to 6 months of therapeutic anticoagulation, clinicians are faced with the complex decision of whether to stop or continue anticoagulation therapy. The challenge lies in balancing the risk of recurrent VTE without extended anticoagulation against the risk of bleeding associated with extended anticoagulation. This decision must be made on a case-by-case basis, taking patient-specific factors into account. Current guidelines recommend discontinuing anticoagulation in patients at low risk of recurrence (eg, provoked by a transient risk factor), and offering extended anticoagulation to patients with unprovoked VTE or VTE provoked by a chronic risk factor, unless there is a high risk of bleeding [[1], [2], [3]].

With the decision to continue anticoagulation comes the question of which anticoagulant and dosing strategy should be used to efficiently prevent recurrent VTE while minimizing bleeding complications. The 2021 American College of Chest Physicians guidelines recommend direct oral anticoagulants (DOACs) over vitamin K antagonists for the secondary prevention of VTE (strong recommendation), and suggest using reduced-dose over full-dose DOACs in this setting (weak recommendation) [2]. However, this last recommendation is based on 2 randomized controlled trials (RCTs) that enrolled patients initially treated for 6 to 12 months and in whom there was equipoise with respect to the continuation or discontinuation of anticoagulation; these trials were neither designed nor powered to compare the efficacy and safety of reduced-dose *vs* full-dose DOACs for extended anticoagulation [4,5].

Recently, results from 3 large-scale RCTs comparing reduced-dose *vs* full-dose DOACs for the secondary prevention of VTE have become available [[6], [7], [8]], providing new insights into the efficacy and safety of these dosing strategies.

## 1 The Clinical Dilemma: Efficacy *VS* Safety for the Secondary Prevention of VTE

In this issue of *Research and Practice in Thrombosis and Haemostasis*, Ahmed et al. [9] attempt to provide a more compelling answer to the question of the risk-to-benefit ratio of reduced-dose DOACs for the secondary prevention of VTE by conducting a systematic review and meta-analysis. Their study is both timely and clinically relevant because it synthesizes data from all RCTs published to date comparing reduced-dose *vs* full-dose DOACs for the extended treatment of VTE, thereby increasing the level of certainty from previous meta-analyses [10]. The authors pooled data from 5 RCTs involving 8781 patients, 4395 of whom (50%) were randomized to receive reduced-dose DOACs. The primary efficacy outcome was objectively confirmed recurrent VTE, and the primary safety outcome was major or clinically relevant nonmajor bleeding (CRNMB), as defined by the International Society on Thrombosis and Haemostasis criteria [11,12]. Secondary outcomes included major bleeding, CRNMB, all-cause mortality, and VTE-related mortality. A random-effects model was used to compare the outcomes associated with different dosing regimens. The strengths of this meta-analysis include the high quality of the included studies, all of which were well-designed multicenter RCTs at low risk of bias, as well as the lack of heterogeneity in the primary outcomes analyzed. Overall, the results demonstrate that reduced-dose DOACs improve the risk-to-benefit ratio of extended anticoagulation by reducing the bleeding risk without compromising efficacy, enabling more definitive conclusions.

## 2 Key Findings: Efficacy and Safety of Reduced-Dose Doacs

One of the most reassuring findings of this meta-analysis is that reduced-dose DOACs were noninferior to full-dose DOACs in preventing recurrent VTE (risk ratio [RR], 0.94; 95% CI, 0.68-1.29, moderate certainty of evidence). This is consistent with previous studies suggesting that the risk of recurrent VTE decreases over time, and that lower-intensity anticoagulation may be sufficient for secondary prevention in many patients. Therefore, a reduced dose of DOACs is an effective option for the secondary prevention of VTE in stable patients who have completed at least 3 to 6 months of full-dose anticoagulation.

The most compelling finding of this meta-analysis is that reduced-dose DOACs significantly decreased the risk of major or CRNMB (RR, 0.71; 95% CI, 0.61-0.82, high certainty of evidence), major bleeding (RR, 0.62; 95% CI, 0.42-0.92, high certainty of evidence), and CRNMB (RR, 0.75; 95% CI, 0.63-0.88, high certainty of evidence). The 38% reduction in major bleeding is particularly noteworthy since it is the most feared complication of anticoagulation therapy and is associated with a case-fatality rate of 7.57% [13]. The 25% reduction in CRNMB is also significant, as these events, though less severe than major bleeding, still impact quality of life and healthcare utilization. This is particularly relevant to frail patients, including the elderly and those with liver or renal failure, thrombocytopenia, anemia, a history of bleeding, or cancer, who are at a higher risk of anticoagulation-associated bleeding and require safer long-term anticoagulation options.

Although the trials included in the pooled analysis enrolled diverse patient populations (Reduced Dose Versus Full-dose of Direct Oral Anticoagulant After Unprovoked Venous Thromboembolism [RENOVE] [6] excluded cancer patients, while Extending Venous Thromboembolism Secondary Prevention with Apixaban in Cancer Patients [EVE] [7] and APIxaban Cancer Associated Thrombosis [API-CAT] [8] exclusively enrolled patients with active cancer), stratified subgroup analyses found no significant differences between noncancer and cancer patients, suggesting that the benefits of dose reduction may be broadly applicable. This is particularly important given the unique challenges faced by cancer patients, who are more susceptible to bleeding complications due to multiple comorbidities, invasive procedures, or potential drug–drug interactions with anticancer therapies. Although subgroup analyses according to age and renal function were not performed, the elderly and those with renal failure, who are particularly vulnerable, are expected to experience the greatest net clinical benefit from dose reduction [14,15].

## 3 Limitations and Areas for Future Research

These results are encouraging, but several important considerations warrant discussion.

First, only 2 factor Xa inhibitors, rivaroxaban and apixaban, have been evaluated and approved at reduced doses (10 mg daily for rivaroxaban and 2.5 mg twice daily for apixaban) for the secondary prevention of VTE. It remains unclear whether the findings of this meta-analysis can be extrapolated to other DOACs. Additionally, there are no head-to-head comparisons of reduced-dose rivaroxaban *vs* reduced-dose apixaban. The question of whether the 2 drugs differ in terms of efficacy and safety remains unanswered.

Second, the meta-analysis only included RCTs with stringent eligibility criteria, which does not reflect clinical practice. Therefore, the findings cannot be generalized to subgroups of patients who were excluded from or underrepresented in the included trials. These subgroups include women with HERDOO2 scores of 1 or less, patients with antiphospholipid syndrome, patients requiring full-dose anticoagulation for reasons other than VTE, and cancer patients for whom low-molecular-weight heparin remains the preferred option due to a higher risk of bleeding (brain cancers or unresected gastrointestinal or genitourinary cancers), uncertain drug absorption, or potentially relevant drug–drug interactions [[16], [17], [18]]. Caution is also probably warranted for obese patients. In the RENOVE trial [6], reduced-dose DOACs resulted in an increased risk of recurrent VTE in patients with a body mass index ≥ 30 kg/m^2^ (adjusted hazard ratio, 6.72; 95% CI, 0.99-6.72), without improvement in net clinical benefit (adjusted hazard ratio, 0.97, 95% CI, 0.63-1.50). Further research is needed to determine whether these patients should remain on full-dose DOACs.

Third, the follow-up duration for extended anticoagulation was 12 months in all of the included studies, except for the RENOVE trial [6], which had a 5-year follow-up period. The long-term efficacy and safety of reduced-dose DOACs beyond 12 months remain unclear. Real-world studies are needed to validate these results and to better understand the long-term implications of dose reduction strategies over longer treatment periods.

## 4 Conclusions

Over the past decade, DOACs have revolutionized the acute treatment and secondary prevention of VTE by offering convenient oral regimens and reduced doses, respectively. However, the journey toward optimal extended anticoagulant therapy is far from over, and we have much to learn from future research. Meanwhile, studies, such as the one by Ahmed et al. [9] provide valuable information by showing that reduced-dose DOACs are safer and as effective as the full-dose regimen in many patients. This reminds us that sometimes less can be more. These results have implications that extend beyond individual patient care. Reduced bleeding complications could result in fewer transfusions, fewer hospitalizations, lower healthcare costs, and improved quality of life for the millions of patients with VTE who require extended anticoagulation worldwide.

A patient-centered approach that pays particular attention to thrombotic and bleeding risk factors is necessary for making nuanced, individualized decisions about extended anticoagulation. Clinical judgment remains paramount. Most patients with a strong indication for extended anticoagulant therapy may be eligible to receive reduced-dose DOACs for the secondary prevention of VTE. However, patients at a very high risk of recurrent VTE and those requiring full-dose anticoagulation for reasons other than VTE (eg, atrial fibrillation) should continue to benefit from full-dose DOACs. Conversely, continuation of anticoagulation, even at a reduced dose, should be discouraged in patients at a very high risk of bleeding. Patients should participate in the decision-making process, focusing discussions on the tradeoffs between efficacy and safety.

The challenge now is to translate this evidence into clinical practice. It is critical to ensure that the benefits of optimized dosing strategies are reached by the patients who stand to benefit most.
